# Mothers’ and Fathers’ Personality, Infants’ Anger Proneness, and Responsive Parenting

**DOI:** 10.1007/s10826-025-03195-9

**Published:** 2025-10-31

**Authors:** Grazyna Kochanska, Lilly C. Bendel-Stenzel, Danming An

**Affiliations:** 1 Department of Psychological and Brain Sciences, The University of Iowa, Iowa City, IA USA; 2 Department of Psychology, Lehigh University, Bethlehem, PA USA

**Keywords:** Personality, Parenting, Mothers, Fathers, Infant temperament

## Abstract

Parenting in infancy is immensely important for children’s development and therefore, contributors to varying quality of early parenting have been extensively studied. Among those, parents’ personality and its links with parenting have attracted intense interest, but factors that may affect those associations, including characteristics of the child, particularly in father-infant relationships, remain poorly understood (Taraban & Shaw, 2018). We present a study of 200 families, including infants, mothers, and fathers (Children and Parents Study). Parents reported their Big Five traits and distress/psychopathology. We observed their parenting (a composite of responsiveness, positive affect, and reversed negative affect, defined as positive responsiveness) toward the infant in home interactions. The infants’ anger proneness, most often considered a characteristic that poses parenting challenges, was observed in standard temperament episodes, and modeled as a moderator of personality — parenting relations. Mothers showed more responsiveness, more positive affect, and less negative affect than fathers. Fathers’ higher Agreeableness, Openness, and Extraversion were associated with more responsiveness. The effect of Agreeableness was further qualified by its interaction with child anger proneness: More agreeable fathers were more responsive toward infants high or average in anger proneness, and highly disagreeable fathers were less responsive toward those infants. Mothers’ personality traits, alone or in interaction with infants’ anger proneness, were unrelated to their parenting; however, mothers who were higher in distress/psychopathology were less responsive toward their infants, especially when the infants were high or average in anger proneness.

## Highlights

In a study of a community sample in U.S. Midwest (*N* *=* 200), we examined links between mothers’ and fathers’ self-reported characteristics (personality traits and distress/psychopathology) and their observed positive responsiveness toward their infants, and we tested if those links were moderated by the infant’s observed anger proneness.

Fathers with higher scores on traits of Agreeableness, Openness, and Extraversion were more responsive. Highly agreeable fathers were also more responsive to highly challenging, anger-prone infants.

Mothers with higher scores on distress/psychopathology were less responsive, especially to anger-prone infants.

The study highlights interplay between parents’ and infants’ contributions to their emerging relationship during a key early developmental period.

Responsive, sensitive parent-infant interactions, infused with positive emotion toward the child in the first year of life are uniformly considered a critical foundation for children’s future development, supported by attachment theory (Ainsworth et al., 1971, Thompson, 2006) and socialization theory (Lansford, 2022; Maccoby & Martin, 1983). Voluminous research has emphasized a set of inter-related key qualities that include parental prompt, sensitive, accurate responsiveness to the infant’s cues, along with the related construct of positive emotions (delight, warmth, smiling) as core dimensions of the early parent–child relationship promoting adaptive development (Aksan et al., 2006; Bornstein et al., 2008; De Wolff & van IJzendoorn, 1997;  Dix, 1991; Kochanska, 1997;  Landry et al., 2006; Leerkes et al., 2022). Consequently, interest in predictors of individual differences in responsive, affectively positive parenting remains high.

Although personality traits have been long recognized as one of the key factors underlying people’s behavior in multiple life domains (McCrae & Costa, 1996; Roberts et al., 2007), including family relationships (Asendorpf & Wilpers, 1998; Ozer & Benet-Menendez, 2006; Soto, 2019), research on associations between parents’ personality and parenting has a surprisingly short history. Historically, parenting researchers first focused on maternal depression, with early work in the 1970s and 1980s highlighting its risk for parenting (Kochanska et al., 1987; Weissman & Paykel, 1974), and parental psychopathology has remained a significant topic in parenting research (e.g., Cummings & Davies, 1994; Dix & Meunier, 2009; Downey & Coyne, 1990; Goodman, 2020; Wilson & Durbin, 2010). Interest in parental personality conceived more broadly – beyond psychopathology – is typically traced to Belsky’s ( 1984) influential article on determinants of parenting. Subsequent research has produced multiple empirical studies and reviews (e.g., Belsky & Barends, 2002; Belsky & Jaffee, 2006; Clark et al., 2000; Kochanska et al., 2004, 2007; Prinzie et al., 2009; Smith, 2010; Smith et al., 2007).

Recently, Taraban and Shaw (2018) revisited Belsky’s (1984) model in view of three subsequent decades of research and concluded that it had stood the test of time well. Summarizing new work on parental personality as a determinant of parenting, they concluded that parents’ depression and psychopathology, and among the Big Five, Neuroticism, are generally associated with less adaptive parenting. Agreeableness, Extraversion, and Conscientiousness, and Openness are generally linked to more adaptive parenting, although Openness has been rarely studied (Belsky & Barends, 2002; Bornstein et al., 2003; 2011).

Further, Taraban and Shaw (2018) outlined key future directions. They emphasized the pressing need to examine fathers’ personality and parenting, as most of the research to date has involved mothers, a well-known gap in the field, although fathers have been increasingly assuming coparenting role (Volling & Cabrera, 2025). Perhaps most importantly, they called for considering factors that can moderate personality-parenting links, such as child characteristics, and particularly, negative emotionality, often referred to as child difficulty. Although links between parental personality and parenting for children varying in temperament have been studied, the findings remain inconclusive and highly mixed. Some relations have been found for parents of difficult children and others for parents of easy children (Achtergarde et al., 2015; Bradley & Korwyn, 2019; 2023; Clark et al., 2000; Coplan et al., 2009; Gölcük & Berument, 2021; Hong et al., 2015; Karreman et al., 2008; Kochanska et al., 2012; Koenig et al., 2010; Prinzie et al., 2012); some have been found for mothers and some for fathers. Consequently, our understanding of relations among parents’ personality, their parenting, and children’s temperament remains incomplete, and further research is much needed.

In a comprehensive meta-analysis, McCabe ( 2014) argued that although both parental personality and psychopathology influence parenting, their effects, although overlapping, are distinct, and that measures of maternal psychopathology and personality should both be integrated as predictors of parenting, whenever both are available, to discern their effects on parenting. Unfortunately, such studies are very rare, especially in infancy. This view was echoed by Durbin and Hicks (2014), discussing overlap and distinctiveness between personality and psychopathology, and calling for integration of both in research on personality.

We address those questions using data from a study of mothers and fathers and their typically developing infants (Children and Parents Study, CAPS, *N* = 200; see, e.g., An et al., 2022; An & Kochanska, 2022). Child temperament was operationalized as anger proneness and modeled as a moderator of associations between the parents’ personality and parenting. Following arguments by McCabe (2014) and Durbin and Hicks (2014), we also included measures of parents’ distress/psychopathology along with those of personality traits, given that the relevant data were available.

We focused on infancy, a critical time for the parent-child emerging relationship. Parental traits were assessed through self-report, but parenting and child anger proneness were measured through behavioral observations in lengthy naturalistic and standard paradigms. By reducing the shared method variance problem, this approach overcomes a weakness of multiple extant studies that have used parents’ self-reports to measure their personality, parenting, and child characteristics. Based on the existing literature (e.g., Kochanska et al., 2004; Taraban & Shaw, 2018), we broadly expected that high Neuroticism and overall psychological distress/psychopathology would be linked to less affectively positive, less responsive observed parenting, and high Agreeableness, Extraversion, and Conscientiousness – to more positive, responsive parenting. particularly when the infants were highly challenging and anger prone. Given the highly mixed data on the role of child difficult temperament as moderator of those relations, this direction was exploratory.

## Method

### Participants

CAPS involved two-parent families with infants (born in 2017 and 2018), who volunteered in response to advertisements broadly distributed in a U.S. Midwestern area. The demographic data are in Supplementary Table S1. They were community families, screened for eligibility factors (e.g., two parents living together, a typically developing infant who was a biological child), but not for any risk factors. In 20% of families (*n* = 40), one or both parents were not “White Alone” (i.e., non-White race and/or Latino ethnicity).

### Procedures

The data reported in this article were collected when children were 8 months (*N* = 200, 96 girls), during a 2.5- h home session conducted by a female experimenter (E), incorporating naturalistic, scripted observations, and standard paradigms (first hour, mother and infant, second hour, father and infant; the other parent was asked to stay in another part of the house when the target parent was observed). All procedures in the study had been approved by the University of Iowa IRB (CAPS, 201701705) and reviewed annually. At the outset of the home session, E administered the consents to both parents and answered any questions. The sessions were videorecorded for future coding. Multiple teams of coders established reliability using 15% to 20% of cases; frequent realignments followed to prevent observers’ drift. Kappas, weighted kappas, and intra-class correlations (ICCs) were used to compute reliability, as appropriate. Analyses were conducted in SPSS 29 .0 (IBM Corp., 2023).

### Measures

#### Predictors

##### Child anger proneness

The infant’s anger proneness was observed in standard anger episodes from Laboratory Temperament Assessment Battery (Lab-Tab, Goldsmith & Rothbart, 1996): Car Seat (buckling the child in a car seat; one 60-s trial), Arm Restraint (holding down the child’s arms; two 30-s trials), and Toy Retraction (taking away a toy and holding out of reach; three 15-s trials). All episodes were administered by E, with the parent asked to be neutral and uninvolved. Coders rated the child’s bodily, facial, and vocal expressions of anger in 5-s segments. Bodily anger was rated from 0 (*none*), to 4 (*high-intensity struggle*); facial anger, from 0 (*none*), to 3 (*strong expression in all three facial regions*); and vocal anger, from 0 (*none*), to 3 (*full-intensity cry or scream*). The latency to express anger in each trial was also coded. Reliability for anger expressions, kappas, averaged 0.81 for Arm Restraint, 0.76 for Car Seat, and 0.75 for Toy Retraction; ICCs for the latencies to express anger averaged 1.00. Details of coding are in Supplement 2.

We then summed the codes for discrete anger expressions for each trial, reversed the latency score, and averaged across trials for the entire episode. Those raw scores were standardized and aggregated into a score of anger for each episode (Cronbach’s alphas were 0.80, 0.76, and 0.81 for Car Seat, Arm Restraint, and Toy Retraction, respectively). Those scores cohered across episodes, from 0.15 to 0.22, *p*s = 0.002–0.042, and were averaged into an overall composite of the child’s anger proneness (see An & Kochanska, 2022).

##### Parental personality (The Big Five and Distress/Psychopathology)

Parents completed the Five Factor Inventory-3 (NEO-FFI-3, Costa & McCrae, 2010). Cronbach’s alphas for mothers were as follows: Neuroticism (general tendency to experience negative affects), 0.83, Extraversion (tendency to be sociable, assertive, active), 0.83, Conscientiousness (tendency to be planful, organized, strong-willed, and purposeful), 0.86, Agreeableness (tendency to be altruistic, sympathetic, and eager to help others), 0.72, and Openness (active imagination, aesthetic sensitivity, intellectual curiosity, and independence of judgment) 0.78; the respective alphas for fathers were 0.87, 0.84, 0.85, 0.74, and 0.82. Parents rated each item from 0 (*strongly disagree*) to 4 (*strongly agree*). Parents also completed Brief Symptom Inventory-18 (BSI-18, Derogatis, 2001). BSI-18 produces the scores on the dimensions of Somatization (e.g., faintness or dizziness), Depression (e.g., feeling blue), and Anxiety (e.g., feeling tense or keyed up). Items are rated from 0 (*not at all*) to 4 (*extremely)*. Their sum, Global Severity Index (GSI), is considered the best single indicator of distress/psychopathology, and therefore, we selected the GSI for this study (Cronbach’s alphas 0.84 and 0.92 for mothers and fathers, respectively).

#### Parenting outcomes

##### Parental responsiveness

For each parent-child dyad, parental responsiveness was coded in five interaction contexts (35 min): play without toys (5 min), play with one toy (5 min), parent “busy” in the kitchen (10 min), parent feeding the child a snack (10 min), and routine caregiving (changing the child’s clothes and diaper, 5 min). The parent received one overall rating for each context (e.g., snack, play), on a scale from 1 (*highly unresponsive*) to 7 (*highly responsive*). The rating incorporated the classic dimensions (Ainsworth et al., 1971): sensitivity-insensitivity, cooperation-interference, and acceptance-rejection. The coding anchor points were clearly described, with multiple examples. Coding reliability, weighted kappas, ranged from 0.74–0.90. The ratings cohered across the coded contexts; Cronbach’s alphas for mothers, 0.71, and fathers, 0.67, and they were averaged across the contexts into the overall responsiveness score for each parent. Details of coding are in Supplement 2.

##### Parental affect

The parent’s affect was coded in four contexts (30 min; all the above contexts except play without toys). For each 30-s segment, coders coded expressions of positive affect as 0 (*not present*), 1 (*neutral positive mood*), 2 (*discrete positive emotion*), or 3 (*a strong positive emotion*), and of negative affect as 0 (*not present*), 1 (*neutral negative mood*), 2 (*discrete negative emotion*), or 3 (*a strong negative emotion*). Reliability, weighted kappas, ranged from 0.71–0.98. Details of coding are in Supplement 2.

For each context, we then summed (across the coded segments) the values for the positive affect and those for the negative affect. Finally, we averaged positive affect scores and negative affect scores across the four contexts, so that each parent received the composite score for the positive affect and one for the negative affect.

##### Affectively positive responsiveness

For each parent, we standardized and averaged responsiveness, positive affect, and (reversed) negative affect into a score of affectively positive responsiveness, hereon “positive responsiveness” or “responsiveness” (the constituent scores correlated 0.53–0.81 for mothers and 0.46–0.84 for fathers, all *p*s < 0.001). To confirm the data aggregation, we conducted Principal Component Analyses (PCA) with Varimax rotation for the parenting variables. For both mothers and fathers, the PCAs fully supported the aggregation, by recovering just one component, with positive affect and responsiveness loading positively (mothers, 0.92 and 0.79, fathers 0.92, and 0.71) and negative affect loading negatively (mothers, −0.90, fathers, −0.92). For mothers, Eigenvalue was 2.81, 76% of variance; for fathers, Eigenvalue was 2.19, 73 of variance. All descriptive data and differences between mother-child and father-child measures are in Table 1.

**Table 1:** Descriptive Data for All Measures

Predictors		M				SD			*Range*			N	
Child Anger Proneness^1^		0.00				0.53			[-1.44, 1.75]			200	

	*Mothers*						*Fathers*						
	*M*		*SD*	*N*	*Range*		*M*	*SD*		*N*	*Range*		*p*
N	20.87		7.58	198	[4.00, 42.00]		17.45	7.88		199	[0.00, 38.00]		<0.001
E	29.65		6.76	198	[12.00, 45.00]		27.85	7.07		199	[11.00, 43.00]		0.006
C	34.53		7.09	198	[16.00, 48.00]		32.45	6.43		199	[18.00, 46.00]		0.002
A	36.03		5.16	198	[16.00, 46.00]		32.06	5.47		199	[18.00, 45.00]		<0.001
O	31.82		6.27	198	[16.00, 48.00]		31.60	6.50		199	[15.00, 46.00]		0.666
BSI-18 GSI	6.77		8.27	198	[0.00, 47.00]		6.72	8.46		199	[0.00, 44.00]		0.974
Parenting Outcomes													
Responsiveness	5.49		0.44	200	[3.40, 6.40]		5.28	0.52		200	[3.20, 6.40]		<0.001
Positive Affect	21.34		4.49	200	[0.75, 32.25]		18.93	4.67		200	[5.75, 32.00]		<0.001
Negative Affect	2.42		1.96	200	[0.00, 14.50]		2.76	2.02		200	[0.00, 10.50]		<0.036
Positive Responsiveness^2^	0.00		0.87	200	[-4.87, 1.61]		0.00	0.85		200	[-2.67, 1.65]		----

*P* refers to mother vs. father comparison

N = Neuroticism. E = Extraversion. C = Conscientiousness. A = Agreeableness. O = Openness. BSI-18 = Brief Symptom Inventory. GSI = Global Severity Index

^1^Composite of Anger Proneness in Car Seat, Arm Restraint, and Toy Retraction (all standardized)

^2^Composite of Responsiveness, Positive Affect, and reversed Negative Affect (all standardized)

## Results

### Preliminary Analyses

The correlations are in Table 2. Several patterns were parallel for mother- and father-child dyads. For both, Extraversion and Agreeableness were associated with more positive responsiveness, and psychopathology (BSI-18 GSI) with less positive responsiveness. For both, psychopathology was associated positively with Neuroticism, and negatively with Extraversion, Conscientiousness, and Openness; Neuroticism was associated negatively with Extraversion and Conscientiousness; Extraversion and Agreeableness were positively associated; and Openness and Conscientiousness were negatively associated.

**Table 2:** Correlations among the Studied Measures

		Predictors							Parenting Outcome
		Child Anger Proneness^1^	N	E	C	A	O	BSI-18 GSI	Positive Responsiveness^2^
Predictors	Child Anger Proneness^1^	----	0.05	0.01	−0.02	0.10	−0.04	−0.14^*^	0.01
	N	0.04	0.13	−0.38^***^	−0.43^***^	−0.26^***^	0.14	0.50^***^	−0.12
	E	−0.08	−0.32^***^	0.14	0.13	0.20^**^	0.07	−0.17^*^	0.19^**^
	C	0.08	−0.46^***^	0.29^***^	0.09	0.14	−0.17^*^	−0.33^***^	0.11
	A	0.04	−0.09	0.21^**^	0.09	−0.01	0.15^*^	−0.20^**^	0.20^**^
	O	−0.01	0.14^*^	0.10	−0.19^**^	0.17^*^	0.27^***^	0.15^*^	−0.01
	BSI-18 GSI	−0.01	0.59^***^	−0.23^***^	−0.32^***^	−0.07	0.15^*^	0.15^*^	−0.17^*^
Parenting Outcome	Positive Responsiveness^2^	0.02	−0.22^**^	0.24^***^	0.09	0.23^***^	0.21^**^	−0.15^*^	0.34^***^

Data for mother-child dyads above the diagonal, for father-child dyads below the diagonal, and cross-parent correlations on the diagonal

N = Neuroticism. E = Extraversion. C = Conscientiousness. A = Agreeableness. O = Openness. BSI-18 = Brief Symptom Inventory. GSI = Global Severity Index

^*^p < 0.05. ^**^p < 0.01. ^***^p < 0.001

^1^Composite of Anger Proneness in Car Seat, Arm Restraint, and Toy Retraction (all standardized)

^2^Composite of Responsiveness, Positive Affect, and reversed Negative Affect (all standardized)

Several correlations were unique to one parent. For mothers only, child anger proneness was negatively associated with psychopathology, and Agreeableness correlated negatively with Neuroticism and with psychopathology. For fathers only, Neuroticism and Openness correlated with less and more positive responsiveness, respectively. Extraversion and Conscientiousness were positively correlated, and so were Neuroticism and Openness.

Mothers’ and fathers’ responsiveness was positively correlated. There was little evidence of assortative mating with regard to personality traits, with the exception of Openness and psychopathology, whose scores correlated across the two parents. There were several differences in personality traits and parenting: Mothers had significantly higher scores than fathers on Neuroticism, Extraversion, Conscientiousness, and Agreeableness, and they scored higher on responsiveness and positive affect, and lower on negative affect than fathers.

### Main Analyses: Child Anger Proneness, Parents’ Personality, and their Responsiveness

We addressed our questions with hierarchical multiple regressions. We entered child anger proneness in the first step, the main effects of parental personality (the Big Five and psychopathology) in the second step, and their interactions in the third step. All predictors were first standardized (all parental variables were standardized separately for mothers and for fathers). The results for the final step, with all the predictors entered, are in Table 3 (mothers), and Table 4 (fathers).

**Table 3:** Child Anger Proneness, Mothers’ Personality, and their Interactions as Predictors of Mothers’ Positive Responsiveness (Final Step, with All Predictors Entered)

Predictor	*B*	*SE*	*F*	95% CI	*p*
Child Anger Proneness	−0.011	0.119	<1	[−0.246, 0.225]	0.929
N	0.094	0.080	1.40	[−0.063, 0.251]	0.238
E	0.123	0.066	3.50	[−0.007, 0.252]	0.063
C	0.070	0.068	1.06	[−0.064, 0.205]	0.304
A	0.114	0.064	3.16	[−0.013, 0.240]	0.077
O	−0.026	0.062	<1	[−0.149, 0.097]	0.680
BSI-18 GSI	−0.216	0.077	7.96	[−0.367, −0.065]	0.005
N x Child Anger Proneness	0.217	0.158	1.89	[−0.095, 0.529]	0.171
E x Child Anger Proneness	−0.103	0.119	<1	[−0.339, 0.132]	0.388
C x Child Anger Proneness	0.124	0.148	<1	[−0.167, 0.415]	0.401
A x Child Anger Proneness	−0.129	0.126	1.03	[−0.378, 0.121]	0.311
O x Child Anger Proneness	−0.043	0.121	<1	[−0.282, 0.196]	0.721
BSI-18 GSI x Child Anger Proneness	−0.393	0.114	11.98	[−0.618, −0.169]	<0.001

Order of entry was as follows. Step 1: Child Anger Proneness, R^2^ = 0.00, F_ch_ < 1, *p* = 0.876; Step 2: N, E, C, A, O, BSI-18 GSI, R^2^ = 0.08, F_ch_ = 2.77, *p* = 0.013; Step 3, N x Child Anger Proneness, E x Child Anger Proneness, C x Child Anger Proneness, A x Child Anger Proneness, O x Child Anger Proneness, BSI-18 GSI x Child Anger Proneness, R^2^ = 0.16, F_ch_ = 2.86, *p* = 0.011. Overall F(13,184) = 2.67, *p* = 0.002

N = Neuroticism. E = Extraversion. C = Conscientiousness. A = Agreeableness. O = Openness. BSI-18 = Brief Symptom Inventory. GSI = Global Severity Index

**Table 4:** Child Anger Proneness, Fathers’ Personality, and their Interactions as Predictors of Fathers’ Positive Responsiveness (Final Step, with All Predictors Entered)

Predictor	*B*	*SE*	*F*	95% CI	*p*
Child Anger Proneness	0.140	0.114	1.50	[−0.086, 0.366]	0.222
N	−0.129	0.077	2.78	[−0.282, 0.024]	0.097
E	0.161	0.064	6.25	[0.034, 0.287]	0.013
C	−0.054	0.068	<1	[−0.189, 0.080]	0.428
A	0.155	0.060	6.69	[0.037, 0.274]	0.010
O	0.160	0.060	7.20	[0.042, 0.278]	0.008
BSI-18 GSI	−0.040	0.071	<1	[−0.180, 0.100]	0.575
N x Child Anger Proneness	−0.047	0.145	<1	[−0.334, 0.240]	0.746
E x Child Anger Proneness	0.022	0.135	<1	[−0.246, 0.289]	0.873
C x Child Anger Proneness	−0.208	0.126	2.75	[−0.456, 0.040]	0.099
A x Child Anger Proneness	0.308	0.117	6.96	[0.078, 0.539]	0.009
O x Child Anger Proneness	−0.112	0.130	<1	[−0.369, 0.145]	0.390
BSI-18 GSI x Child Anger Proneness	−0.136	0.139	<1	[−0.410, 0.138]	0.328

Order of entry was as follows. Step 1: Child Anger Proneness, R^2^ = 0.00, F_ch_ < 1, *p* = 0.775; Step 2: N, E, C, A, O, BSI-18 GSI, R^2^ = 0.15, F_ch_ = 5.74, *p* < 0.001; Step 3, N x Child Anger Proneness, E x Child Anger Proneness, C x Child Anger Proneness, A x Child Anger Proneness, O x Child Anger Proneness, BSI-18 GSI x Child Anger Proneness, R^2^ = 0.21, F_ch_ = 1.99, *p* = 0.069. Overall F(13,185) = 3.66, *p* < 0.001

N = Neuroticism. E = Extraversion. C = Conscientiousness. A = Agreeableness. O = Openness. BSI-18 = Brief Symptom Inventory. GSI = Global Severity Index

#### Mother-child dyads

**Fig. 1:**
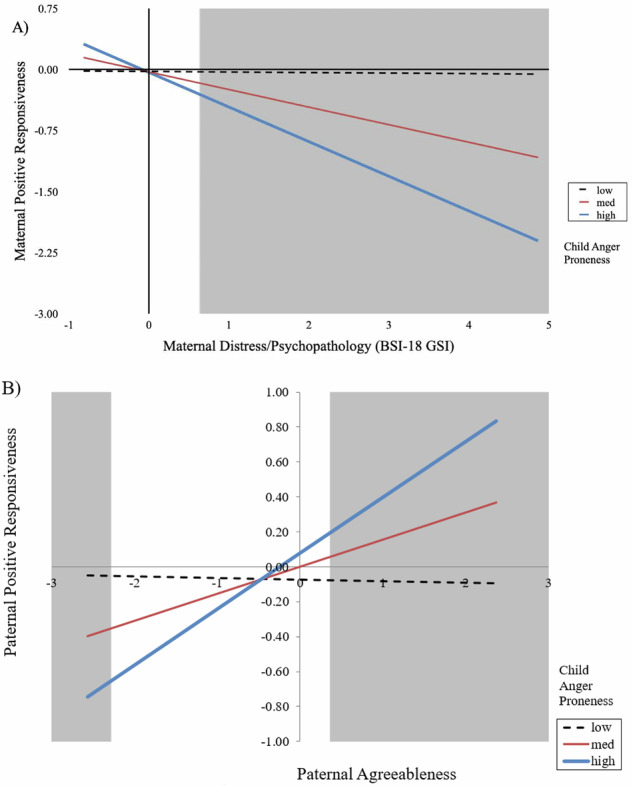
**A**) Children’s anger proneness moderates the effect of mothers’ distress/psychopathology (BSI-18 GSI) on maternal positive responsiveness. **B**) Children’s anger proneness moderates the effect of fathers’ Agreeableness on paternal positive responsiveness. The solid lines represent significant simple slopes, and the dashed lines represent non-significant simple slopes. The shaded areas represent the regions of significance. BSI-18 Brief Symptom Inventory, GSI Global Severity Index

The predictors explained a significant proportion of variance in the final equation, R^2^ = 0.16. The significant effects involved maternal distress/psychopathology: BSI-18 GSI had a main effect: Mothers with higher scores were less responsive. That effect was further qualified, in part, by its interaction with child anger proneness, although it remained significant in the final equation. We probed the interaction using simple slopes (Aiken & West, 1991). The regions of significance (RoS) were determined following recommendations and the strategy proposed by Roisman et al. ( 2012). The results are in Fig. 1A.

Simple slopes indicated that mothers’ higher levels of psychopathology undermined their responsiveness toward infants who were highly anger prone, *B* = −0.425, *SE* = 0.099, *p* < 0.001, and toward infants who were average in anger proneness, *B* = −0.216, *SE* = 0.054, *p* < 0.001. There was no association between maternal psychopathology and responsiveness toward infants who were low in anger proneness, *B* = −0.007, *SE* = 0.059, *p* = 0.912. The inspection of RoS indicated that significant differences in maternal responsiveness toward infants varying in their anger proneness emerged for mothers whose level of psychopathology was above 0.648 *SD* from the mean.

#### Father-child dyads

For fathers, the predictors also explained a significant proportion of variance in the final equation, R^2^ = 0.21. There were three main effects of parental traits: Fathers with higher scores on Extraversion, Agreeableness, and Openness were more responsive to their infants. The effect of Agreeableness was further qualified by an interaction with child anger proneness, such that more agreeable fathers were more responsive toward their infants when the infants were high or average on anger proneness, *B* = 0.319, *SE* = 0.089, *p* < 0.001, and *B* = 0.155, *SE* = 0.063, *p* = 0.015, respectively, as illustrated by simple slopes in Fig. 1B. The level of paternal Agreeableness was unrelated to responsiveness toward infants low in anger proneness, *B* = −0.009, *SE* = 0.089, *p* = 0.916. The inspection of RoS indicated that the differences in paternal responsiveness toward infants varying in their anger proneness were present for both highly agreeable fathers (above 0.337 *SD* from the mean) and highly disagreeable fathers (below −2.28 *SD* from the mean). Note, however, that although calculable, the latter scores should be interpreted with caution, as having limited practical utility, given that only a few fathers were in that low range.

## Discussion

We examined associations between parental personality and parenting in infancy, a key period for the emerging parent-child relationship. Our goals were to address issues explicitly articulated as important future directions in this growing field. We aimed to include mothers and fathers and to examine child characteristics, particularly anger proneness, or “difficult temperament”, as moderating personality-parenting associations. Having a highly emotionally negative or anger-prone infant can be a source of significant stress. Personality scholars have long linked personality traits with strategies people deploy to cope with stress (Carver & Connor-Smith, 2010; Connor-Smith & Flachsbart, 2007; David & Suls, 1999; Watson & Hubbard, 1996), including interpersonal stress, such as dealing with a difficult child (Lee-Baggley et al., 2005). Generally, Agreeableness, Conscientiousness, Openness, and Extraversion have been linked to more adaptive coping strategies, whereas Neuroticism has been linked to less adaptive strategies. That conceptual framework can be particularly relevant and useful for research on parental personality in the context of challenges and stresses associated with raising a temperamentally difficult child (Kochanska et al., 2012).

We also sought to consider parents’ Big Five traits along with their distress/psychopathology, incorporating recent arguments that both sets of measures should be examined if available (Durbin & Hicks, 2014; McCabe, 2014; Taraban & Shaw, 2018). All measures were well established and robust. To assess parental traits and distress/psychopathology, we deployed NEO-FFI (Costa & McCrae, 2010) and BSI-18 (Derogatis, 2001), respectively. 

. We used standard behavioral paradigms to measure infants’ difficulty, or anger proneness (Lab-Tab, Goldsmith & Rothbart, 1996). We observed key parenting dimensions during infancy – responsiveness and positive and negative affect expression – in lengthy naturalistic interactions. Such approach eliminates weaknesses associated with designs that utilize parents’ reports of their personality, parenting, and the child’s characteristics. The findings revealed both main effects of parental personality on parenting and the role of child temperament as a contextual moderator of those associations.

Although we were interested mostly in associations among parental traits, child temperament, and parenting, and used standardized scores (separately for mothers and fathers), we note additional findings before standardization, indicating gender differences in Neuroticism, Extraversion, Conscientiousness, and Agreeableness (all higher for mothers). Those are largely consistent with the extant reports (e.g., Weisberg et al., 2011). It was interesting that we found no differences in distress/psychopathology, often reported to be higher for women.

The current work produced little evidence of evocative effects of child temperament on parenting (i.e., parents’ being more or less responsive to their infant as a function of the infant’s temperament). This is consistent with the growing recognition that effects of child temperament on parenting are complex and can be moderated by multiple factors (Kochanska & Kim, 2012; Paulussen-Hoogeboom et al., 2007). We did, however, find that infants’ temperament served as a moderator of associations between parents’ characteristics and child temperament.

### Findings for Mothers and Children

Mothers were more responsive, more positive, and less negative in their interactions with the infants. This finding is consistent with a recent meta-analysis (Deneault et al., 2022). We found few significant relations among mothers’ characteristics, their parenting, and infants’ temperament. There were no effects involving the mothers’ Big Five, but there were effects associated with psychological distress/psychopathology. 

We found that for mothers, overall distress/psychopathology, assessed in BSI-18, added unique variance beyond the Big Five when predicting parenting. Mothers with higher scores were less responsive, both as a main effect and in interaction with infant difficult temperament, being less responsive to infants who were high or even average in anger proneness. Mothers with high distress/psychopathology scores, who may have been especially affected by their infant’s intense anger, showed relatively less positive responsiveness, consistent with the literature (Bradley & Corwyn, 2023; Carver & Connor-Smith, 2010). We note that although the scores of distress were in general quite low, as expected in a community sample, its effect was nevertheless quite robust.

Our findings supported McCabe’s ( 2014) argument that, in research on parenting (and more generally, on personality, Durbin & Hicks, 2014), whenever available, personality traits and psychopathology should both be examined together, as their roles can be distinct. Further, personality traits are likely to be quite stable, whereas feelings of distress can be due both to dispositional factors and current stressors and adverse circumstances, thus more variable over time than personality traits. This is one reason why personality traits and psychopathology may have different impacts on parenting.

### Findings for Fathers and Children

There were more findings for personality-parenting links for fathers than for mothers, supporting the importance of research addressing the stubborn gap in developmental psychology, still predominantly focused on mothers and young children (Parke, 2000; Volling & Cabrera, 2025 ). Elucidating determinants of individual differences in early fathering is critically important, as has been increasingly stressed (Bakermans-Kranenburg et al., 2019; 2023). The findings for fathers involved three traits: Agreeableness, Openness, and Extraversion. Fathers with higher scores were more responsive than fathers with lower scores. High Agreeableness and Extraversion have been repeatedly associated with warm, positive parenting; however, the findings for Openness are notable, because that trait has rarely been studied. Persons who score high on Openness are imaginative, open-minded, open to new experiences, intellectually curious, and independent in their judgment, and have broad interests. We interpret our findings as possibly indicating that highly open fathers may be more accepting of the relatively nontraditional role as a caregiver of a young infant – one historically seen as associated with mothers – and that they function well in that role.

Highly agreeable fathers were more responsive when their infants were either high or average in anger proneness, again consistent with the literature on personality and coping (Carver & Connor-Smith, 2010). When confronted with the stress of having a challenging infant, highly agreeable fathers may be less likely to disengage and withdraw, but instead, more likely to focus on the infant’s needs and more able to recruit behaviors and emotions associated with trait Agreeableness: positive affect, warmth, empathy, comfort, and support needed to calm the child down. Further, we also found that infants’ anger proneness was associated with paternal responsiveness among highly disagreeable fathers, such that those fathers were especially unresponsive to anger-prone, difficult infants. Although few fathers in our study fell in the very low range of scores, note that simple slopes refer to predicted rather than observed values; consequently, they indicate a possibility of highly dysfunctional parenting for very disagreeable fathers, whose infants are also highly anger prone, for example, in certain clinical samples.

It was interesting that we did not find effects of paternal distress/psychopathology or its interaction with infants’ difficult temperament for fathers. Likely, the amount of stress experienced when parenting a difficult infant was greater for mothers than for fathers, as mothers spent more time caring for the child (on average 59 vs. 35 h per week, *p* < 0.001). And yet, recall that mothers were more responsive and positive overall, compared to fathers.

### Future Directions

Although studies of personality-parenting links, as in the current work, are important, they should be seen as a starting point for the next generation of research. That future research will need to address cognitive and affective processes, or mediators, that account for those empirical associations (Belsky & Jaffee, 2006; Taraban & Shaw, 2018). That work could perhaps draw inspiration from frameworks proposed in research on parental depression (Dix et al., 2004; Dix & Meunier, 2009). Several such mediating processes have been proposed, such as parenting cognitions (Bornstein et al., 2003; 2011), the sense of competence (de Haan et al., 2009), and the parent’s moods (Belsky et al., 1995) and emotion regulation capacities (Dix & Meunier, 2009). Other processes may potentially also serve as such links, for example, parental self-regulatory and executive function skills (Bridgett et al., 2015; Jones-Gordils et al., 2021), which have been strongly associated with Conscientiousness in personality psychology (Roberts et al., 2014). Longitudinal designs are best suited to examine such links, with the specific processes modeled as the mediating mechanisms. In our past preliminary work with the CAPS data, we tested and supported the role of parents’ explicit and implicit internal working models – or representations – of the child as such mediators, but we only examined two of the Big Five – parental Neuroticism and Agreeableness (An et al., 2022). We will continue to study this issue as we follow this sample. Such research will not only provide new insights that will integrate research on parenting and personality, but it will also elucidate potential windows for new parenting interventions.

Future work should also situate parental personality research in family systems perspectives (Belsky, 1984; Cowan et al., 2019; Harold & Sellers, 2018; Taraban & Shaw, 2018). Parenting is influenced by harmonious or adversarial inter-parental relationships; and parental personality likely impacts both. As well, other factors in families’ ecologies ought to be considered along with parents’ and children’s characteristics, including community support, employment, life stress, and sociodemographic resources and adversities (Bornstein et al., 2003; Kim & Kochanska, 2021).

### Practical Implications

Although we studied community families, in which parents were generally responsive and positive, and reported little psychological distress, the findings may have practical implications, as the effects may be amplified in families experiencing more stress and life challenges. Thus, this study has a potential to inform future early parenting intervention and prevention programs. As one example, parents, especially mothers, who experience high levels of psychological distress, either due to trait-like disposition or to temporary state-like stressors, and who also have temperamentally challenging infants, should be targeted for support. Several studies have indicated that a combination of maternal distress and infant irritability is associated with developmental risks that may increase over time (e.g., Wiggins et al., 2014). Importantly, interventions for parents with difficult, irritable infants have been found to be especially successful, both in classic (van den Boom, 1994) and recent studies (Cassidy et al., 2011). Further, given the increasingly recognized importance of studying fathers as early caregivers (Volling & Cabrera, 2025), our data provide significant leads for understanding paternal personality as a source of individual differences in their parenting in the first year of life.

### Strengths and Limitations

The strengths of this work include a relatively large sample and a multi-method approach that combined established parental self-reports with extensive and labor-intensive behavioral measures of parenting and infants’ temperament. Parallel data from mother- and father-infant dyads address a key gap in the field.

This study has limitations. To focus on infancy, a key period in the emerging parent-child relationship, we conducted only one assessment in the first year, and thus all measures were concurrent. However, one can reasonably assume that the parents entered the relationships with their infants having their own personalities already formed. As well, although there is a broad consensus that in longitudinal studies, relations between child characteristics and parenting are bidirectional, in our model, child anger proneness was considered a moderator of personality-parenting link;  thus, the fact that its assessment was concurrent should not be problematic.

The first assessment of the child’s difficulty or anger proneness in CAPS was conducted at 8 months of age. Although we considered those measures to reflect a “child factor” and we examined how it moderated the links between parents’ personality traits and parenting, it is important to note that earlier parental care, during the first months of life, contributes to children’s early emotionality, including emotional regulatory and reactive processes (e.g., Keenan, 2000; Propper & Moore, 2006; Swingler et al., 2014). Ideally, measures of individual differences in infant psychophysiology and parental care, and their associations, should both be collected throughout infancy.

We focused on the infants’ anger proneness, a characteristic that has been by far the most often studied as a salient parenting challenge (e.g., Slagt et al., 2016; Taraban & Shaw, 2018; van Zeijl et al, 2007) and one that can be robustly assessed in infancy. However, a more complete understanding of children’s temperament as a moderator of effects of parental personality on parenting will require expanding the repertoire of the studied temperament characteristics (Kiff et al., 2011). Those may encompass various traits reflecting positive and negative reactivity, becoming more differentiated as children mature (Rothbart & Bates, 2006). For example, in studies with older children, shyness and emotional dysregulation have moderated links between parental personality and coercive, authoritative, and over-protective parenting, as reported by mothers of 6-year-old children (Coplan et al., 2009).

Likewise, when assessing parental personality, we focused on the Big Five, again by far the most often studied in the extant literature. However, including more comprehensive personality assessments will foster progress toward a better understanding of personality-parenting links. The extant research examining parental traits beyond the Big Five suggests that empathy (Clark et al., 2000), socialization (Kochanska et al., 2007), optimism (Koenig et al., 2010), or behavioral activation and inhibition (Bailes et al., 2024) may be important as well.

It will also be useful to examine parenting in a more nuanced manner. We focused on a composite of responsiveness and positive and negative affect, as they clearly converged empirically, and all have been long considered inter-related (e.g., De Wolff & van IJzendoorn, 1997). Due to standardization, each contributed approximately equally to the composite. However, examining those dimensions separately, and before standardization, might lead to new insights.

The participants came from two-parent community families, with typically developing infants, and with limited racial and ethnic diversity (although well representative of our state’s demographics). The parents were overall responsive and affectively positive; negative affect was infrequent. Their overall distress/psychopathology scores in BSI-18 were low, common in non-clinical populations. In future work, including samples enriched or selected for features such as dysfunctional personality traits (e.g., personality disorders) and elevated psychopathology and distress, challenging and stressful family relationships (e.g., abuse, neglect, chaos, discord, violence), or contextual adversity (e.g., low income, unemployment, dangerous neighborhood, single parenting, absence of social support) would be informative. Of note, we have examined links between mothers’ Big Five and their parenting in another sample of highly stressed, low-income, diverse mothers of toddlers (Kochanska et al., 2012) and found that not all associations were the same as in community families: Mothers high on Openness were more responsive to their less difficult children rather than to their more difficult children. However, children were older and their difficulty was assessed using different methods than standard anger episodes, so that study is not directly comparable with CAPS.

Despite the limitations, the findings contribute to the growing knowledge of early determinants of parenting, and especially, differences between mother- and father-child emerging relationships. As often reported (Deneault et al., 2022), mothers were more responsive and more affectively positive as caregivers of their infants. Their personality traits were unrelated to the quality of their parenting, somewhat in contrast to several earlier studies. However, their level of psychological distress was associated with less responsive parenting, especially toward difficult infants. On the other hand, fathers’ personality traits were associated with their parenting: Fathers who were highly extraverted, agreeable, and open were more responsive and positive when interacting with their infants. Further research on the interplay among parental personality, their early caregiving, and children’s temperament characteristics promises to remain an exciting enterprise.

## Supplementary information

Table S1

Supplement 2

## Data Availability

Although we will gladly share the coding systems or the syntax used in the analyses, we are unable to share data for individual families. Our consent forms the parents signed as they entered each study clearly preclude any sharing of individual data, even if deidentified. The parents have consented to the data being shared in the aggregate form only, and we are ethically and legally bound to follow this agreement.
