# Supplementary material for: Mothers’ and Fathers’ Personality, Infants’ Anger Proneness, and Responsive Parenting
Source: J Child Fam Stud. 2025 Oct 31;34(12):3302–14. doi: 10.1007/s10826-025-03195-9 (PMC12695959; doi:10.1007/s10826-025-03195-9)
Supplement: Supplementary file 2 — Supplement 2 [file 10826_2025_3195_MOESM2_ESM.docx]

**Mothers’ and fathers’ personality, infants’ anger proneness, and responsive parenting**

**Supplement 2: Coding Systems**

**PARENTAL RESPONSIVENESS CODING SYSTEM (4 CODERS)**

Begin coding each paradigm when E says ‘start’ or closes the door.

Paradigms Coded (same for both mother and father sessions):

Context Duration

Play with No Toys 5 min

Play with One Toy 5 min

Mom/Dad Busy 10 min

Snack 10 min

Caregiving 5 min

Total 35 min

Each paradigm is coded for the parent’s responsiveness along the global conditions described below. **One overall rating** (1-7) is given based upon three scales of global responsiveness: Sensitivity/Insensitivity, Cooperation/Interference, and Acceptance/ Rejection. Generally, high scores are given when the parent’s behavior is likely to please the child.

Sensitivity - Insensitivity

This scale describes the amount and quality of the attention a parent gives her/his child. It refers to how aware the parent is of the child's needs/feelings/whereabouts, and how promptly and appropriately the parent responds to the child's cues or signals. It also involves the extent to which the child's needs are met by the parent.

Cooperation‑Interference

This scale describes a parent's respect for the child as an autonomous individual with his/her own wishes and desires. Although a child's wishes and desires must not be always abided, a cooperative parent allows the child a **moderate amount of autonomy, appropriate to the circumstances.** The extent of how controlling the parent appears, either **physically or emotionally**, is a determinant of a parent's score on this scale.

Acceptance‑Rejection

This scale describes how **genuine versus perfunctory a parent's enjoyment and interest is** when interacting with the child. It also describes how much attention the parent gives to competing concerns when interacting with the child. Third, it describes how much the parent seems to enjoy his/her interactions with the child.

**7 (Highly Responsive)**

The highly sensitive parent is one who is very aware of the child and his/her needs. When the child signals for attention, parent acts promptly and appropriately to attend to the child, correctly interpreting the child's signal. The highly sensitive parent is continually "in tune" with the situation surrounding the child, perceives the possibility of trouble, and redirects the situation appropriately. Parent has an "emotional presence" with the child. Offers attention spontaneously, even if the child does not solicit it.

A highly sensitive parent:

- Consistently watches to make sure the child is O.K.
- Always quickly and appropriately responds to child's verbal and nonverbal bids (sneezes, falling down, etc).
- Can and does anticipate child's needs without interfering or hindering child’s autonomy.
- Is capable of engaging child, and uses this ability to make tasks more enjoyable (specific to the task at hand)
- Senses and perceives things that the child enjoys or wants (such as tickling, singing, etc.), and responds appropriately.
- Validates child's wants and desires even if he or she ultimately needs to restrict the child's behavior.
- ALWAYS understands child=s signals/verbalizations and responds appropriately.
- Definitely has an *emotional presence* with his/her child throughout paradigm; is *in tune* with child.
- Offers much spontaneous attention to the child, in line with the task of the paradigm.

The highly cooperative parent is one who acknowledges that the child is an autonomous person, deserving of respect as an individual.

A highly cooperative parent:

- Modifies agenda during activities, especially those which require competitive attention (i.e., Mom busy with questionnaires), based on cues from child.
- Follows child's lead whenever appropriate to do so.
- When child engages in exploratory behavior, encourages this behavior when not inappropriate or dangerous.
- Does not use direct commands, but gives the child ideas instead.

The highly accepting parent is one who displays a very genuine interest in the child and seems to genuinely enjoy interacting with him or her. The child is not a cause of frustration. His/her acceptance of the child is evident and unwavering. Parent appears to have a good time with the child, even when things do not go smoothly or harmoniously.

A highly accepting parent:

- Appears to truly be having fun when interacting with child (laughs, smiles).
- Verbally and affectively, does not seem express frustration when things do not go well or child is uncooperative.
- Appropriately (firmly) corrects behavior when necessary, but this does not linger.
- Often makes positive comments about the child's behaviors that indicate an accepting attitude.
- Transfers his/her excitement, affection, and/or love and enthusiasm to the child.

**6 (Responsive)**

The sensitive parent is one who is often aware of the child and his/her needs but doesn't anticipate his/her needs. When the child signals for attention, parent acts relatively promptly and appropriately to attend to the child, correctly interpreting the child's signal. The sensitive parent is often "in tune" with the situation surrounding the child. Most of the time parent can perceive the possibility of trouble and redirect the situation appropriately. Most of the time parent has an "emotional presence" with his/her child. Sometimes parent offers attention spontaneously, even if the child does not solicit it.

A sensitive parent:

- Frequently watches to be sure the child is O.K.
- Frequently is prompt in responding to the child's bids.
- Frequently interacts with child
- Often senses and perceives things that the child enjoys or wants (such as tickling, singing, etc.), and responds appropriately.
- Sometimes validates the child's wants and desires even if he/she ultimately needs to restrict the child's behavior.

The cooperative parent is one who sometimes acknowledges that the child is an autonomous person, deserving of respect as an individual. The parent sometimes considers the child's desires and feelings, within reasonable limits.

A cooperative parent:

- Frequently modifies agenda during activities based on cues from child.
- Allows for significantly more cooperation than interference.
- When it is necessary to physically redirect the child, does so in a gentle and guiding way.

The accepting parent is one who, most of the time, displays a genuine interest in his/her child and seems to genuinely enjoy interacting with him or her. Parent does not appear to get frustrated easily by child. Parent appears to have a good time with the child most of the time.

An accepting parent:

- Appears to enjoy interacting with the child (some positive affect must be present).
- Does not express frustration when things do not go well or child is uncooperative (verbally and/or affectively).
- Often makes positive comments about the child's behavior that indicate an accepting attitude (a large percentage of comments made are positive to/about the child).

**5 (Somewhat Responsive)**

A somewhat responsive parent is one who sometimes has an emotional presence with the child and appears to be “in tune” some of the time.

A sensitive parent:

- Tends to respond to the child's verbal cues but not to his or her non-verbal (e.g., physiological) cues.
- May respond to child but does not give spontaneous attention
- Does not always respond appropriately (includes “automated” responses, such as “really”, “uh-huh”, etc).

A cooperative parent:

- A little more cooperation than interference
- Open-ended commands
- Allows child to take some of the lead within the limits of the set agenda
- Allows, but may not encourage, exploratory behavior.

A somewhat accepting parent is generally content to be with the child, although may not express this overtly as much as more accepting parents.

An accepting parent:

- Is having a little more fun than if was alone.
- Expresses warmth in majority of interactions with child.
- Expresses little to no frustration, and if displayed it is only at appropriate times.
- Makes genuine positive comments to/about the child.

# **X THERE IS NO 4**

# **3 (Somewhat Unresponsive)**

The insensitive (fair) parent is one who is sometimes unaware of the child and his/her needs. When the child signals for attention, parent acts relatively slowly and often inappropriately to attend to the infant, sometimes misinterpreting the child's signal. The insensitive parent is not really "in tune" with the situation surrounding the child. Sometimes parent can perceive the possibility of trouble and redirect the situation appropriately, but not usually. Often does not have an "emotional presence" with his/her child.

A somewhat insensitive parent:

- May inconsistently watch to make sure the child is O.K.
- Slowly responds to child's bids.
- Rarely talking to child.
- Doesn't respond to child's overt non-verbal (e.g., physiological) cues.
- May often respond but often not appropriately.
- If parent consistently watches child, does so in a neutral/non-interactive manner.

The interfering parent does not appear to have much respect for the child's individuality or autonomy. Oftentimes, the parent's agenda takes precedence, although he or she may deviate from it occasionally. Interference may also take the form of physical interference with the child's activities, either by physically redirecting the child's behavior or by reorganizing the situation, without concern for the child's preference.

An interfering parent:

- Often chooses toys/ games, despite cues from child that he/she is not interested.
- Often restricts child's exploratory behavior when it does not comply with the PARENT’S agenda (not the task agenda, such as cleanup).
- Uses many direct commands
- Somewhat equal amounts of cooperation and interference
- Does not follow child's lead
- May seldom modify agenda
- Sometimes allows exploratory behavior, even when this behavior is marginally inappropriate or dangerous.
- When physically redirecting child, may do so in a controlling manner.

The rejecting parent appears to be wishing to be somewhere else at least for part of the time. Parent does not show enthusiasm and may be easily frustrated by the child or impatient with the child.

A rejecting parent:

- Does not appear to be having a good time with the child (flat/neutral affect).
- Sometimes even positive verbal statements may be made in a tone indicating disapproval or negative affect.
- Gets mildly upset, impatient, and/or irritable when child becomes difficult, unenjoyable, or less cooperative.
- Would have as much fun if alone.
- Rarely makes genuine positive comments to/about child.

**2 (Unresponsive)**

An insensitive parent:

- Discounts the child’s bids
- Not “in tune” to child’s situation, wants, desires, etc.
- Doesn’t make an effort to choose things that the child wants.
- Rarely monitors child’s whereabouts.
- Doesn’t validate child’s wants and desires when restricting behavior.

An interfering parent:

- Seldom follows child's lead
- Uses more interference than cooperation.
- Allows for very inappropriate/a lot of inappropriate behavior.
- Shows little regard for child's preferences (when child shows clear preference).
- When physically redirecting child, may do so in a harsh manner.
- Parent’s agenda takes precedence.

A rejecting parent:

- Would be happier if alone.
- Is often critical of his/her child, and may make critical or rejecting comments directly to child.
- May show frustration or impatience, even when not warranted.
- Appears disinterested in child.
- Would have more fun if alone

**1 (Highly Unresponsive)**

The highly insensitive parent is not concerned as much with the situation of the child as with his/her own situation and agenda. If parent responds to the signals of the child, is likely to do so in a way inconsistent and inappropriate to the meaning of the child's signal.

A highly insensitive parent:

- Is more likely to respond to the negative behaviors when the child displays both positive and negative behaviors.
- Pays little to no attention to the child when involved in other activities, little monitoring of whereabouts.
- Often does not respond verbally or otherwise to child's cues (both verbal and nonverbal cues). More often response time is really slow.
- Is unresponsive and uninvolved during activities, does not attempt to make activities "fun"

The highly interfering parent does not appear to have any respect for his/her child's individuality or autonomy. Oftentimes, the parent's agenda takes precedence, and is the only acceptable agenda to follow. The highly interfering parent allows little deviation from the own agenda or plan. Interference may also take the form of physical interference with the child's activities, either by physically redirecting the child's behavior or by reorganizing the situation, without concern for the child's preference.

The highly interfering parent:

- Always chooses toys/ games, despite cues from child that he/she is not interested.
- Shows no desire to modify agenda, even during play.
- Completes tasks with little regard for child's preferences. May put food in child's mouth before he/she seems ready, or delay until parent is ready.
- Is quick to restrict child's exploratory behavior when it does not comply with his/her agenda.
- Discounts child's desires.
- Treats child like an inanimate object (i.e., physically moves child's limb to perform the behavior desired by the parent when unnecessary).

The highly rejecting parent appears to be wishing to be somewhere else. He or she does not show enthusiasm and is easily frustrated by his/her child.

A highly rejecting parent:

- Is easily upset, impatient, and/or irritable when child becomes difficult, unenjoyable, or less cooperative. May make verbal statements indicating disapproval of child, and may direct them to the child.
- Ignores child, or sighs in a way that indicates he or she is not interested in the child, or is frustrated with interactions with him/ her.
- Shows high frustration/impatience.

**PARENTAL AFFECT CODING SYSTEM (4 CODERS)**

Coders view videos of various paradigms and code parent and child affect. Facial expressions, body language, and tone of voice are all indicators of affect. The coding is done for each 30-second segment. Different paradigms will vary in their length, and have standard numbers of segments. If a paradigm runs over time, some segments will not get coded. If a paradigm is somewhat shorter than it should be, then some segments will remain blank. If the last segment is only partially completed (e.g., 19 seconds rather than 30), coders use whatever is available for that segment. For each segment, the coder assigns a code for positive and negative affect to the parent and to the child (detailed conventions below).

POSSIBLE CODES

*Positive*

0: Not present. 1: Neutral positive mood. 2: Discrete positive affect. 3: Intense positive affect.

*Negative*

0: Not present. 1: Neutral negative mood. 2: Discrete negative affect. 3: Intense negative affect.

CONVENTIONS

If neutral positive (1) or neutral negative (1) is coded, it is the only code (for the respective affect) given to this segment. Then, 0 must be given to the other affect. In other words, for one person, the neutral code (1) may only stand alone in a segment (i.e., no 2 or 3 may be put in that segment). If a code of 2 or 3 is used, that fact precludes, by convention, using neutral (1) in that segment. If neutral positive is entered, enter 0 for negative affect. If neutral negative is entered, enter 0 for positive affect.

If positive or negative affect is intense (3), use only code 3 and not code 2, even if affect escalated during the segment from 2 to 3.

Occasionally, a coder may hesitate whether to use a neutral code (1) or a discrete affect code (2), because some emotion expressions are quite fleeting (especially in infants). If in doubt, use the following hints. In ambiguous cases, if an affect, positive or negative (2), is displayed for at least two seconds, code as discrete affect. Otherwise, neutral (1) is probably a better choice for very fleeting and relatively weak expressions. However, in cases when a discrete positive or negative affect (2) lasts less than two seconds, but is emotion is fully formed (a brief cry, a brief laugh), thus not ambiguous, code as a discrete affect (2).

Both positive affect (2 or 3) and negative affect (2 or 3) may be given in one segment to the same person. For example, a child may laugh and cry in the same segment.

During some paradigms, the parent or child may not be visible, however, their voice may be used to determine the coding category. A parent must be neutral positive for at least half the segment (15 seconds) for that code to be given, otherwise, a neutral negative should be coded. (i.e., the parent must be looking at or interacting with the child for at least half the segment) In the busy context, the parent only needs to be interacting with the child for 7 seconds to receive a neutral positive code.

PARENT POSITIVE AFFECT

*Code 0 – Not Present*

Parent shows no positive affect.

*Code 1 – Neutral Positive*

Parent has a neutral expression with no emotional tint or with a positive tint.

There has to be some degree of animation, generally positive affect expression (even if short of a full smile). Parent appears to be in a good mood and emotionally present; you feel like approaching her/him and beginning an interaction, and you think she/he would respond warmly and eagerly. She/he looks genuinely comfortable and enjoying the “here and now.” In the snack situation, she/he keeps talking to the child, makes overtures that are “bright,” cheerful, and positive in content and vocal quality, even if she/he is busy with getting food ready. She/he may watch the baby with a warm and perhaps half-amused expression, even if she/he is not interacting with her/him.

*Code 2 – Discrete Positive*

Parent exhibits at least one clear occurrence of joy and/or affection (positive affect). Parent smiles, or shows tenderness, affection toward child. Physically or verbally demonstrative, often accompanied by tender, caring tone of voice, kissing (either directly or through other means such as a puppet or through the air), stroking, hugging, tickling, positive comments or reinforcement accompanied by physical tenderness. Parent smiles or laughs briefly.

*Code 3 – Intense Positive*

Any discrete affect that persists for more than 15 seconds (cumulatively) or affect that occurs several times in one segment (thus, most likely accumulates to more than 15 seconds) should be coded. Any affect that is particularly intense, affect that clearly escalates, or affect that is pervasive and intense should be coded. Enthusiasm, sustained or repeated laughter, giggling, strong display of affection. Joy and/or affection expressed in multiple channels (face - full smile, voice – singing, laughing, exaggerated baby talk, body – swinging arms, hugging baby, waving toy or baby playfully, kissing baby, body-to-body contact with baby).

PARENT NEGATIVE AFFECT

*Code 0 – Not Present*

Parent shows no negative affect.

*Code 1 – Neutral Negative*

Neutral expression with a negative tint, a touch of impatience, creates an impression that she/he “would rather be elsewhere.” Appears not fully emotionally present. You feel that, if given a choice, the parent would much rather be elsewhere, or that she/he would be glad and relieved if the session were over. There may be subtle signs of fatigue, impatience, or worry. You have a feeling that she/he is participating out of the sense of duty, “going through the motions,” putting up with the child and requirements of the session, and not having a jolly good time, although, of course, the experience is not clearly unpleasant, either. The situations when neutral negative are most likely are those in the living room. In the snack situation, code neutral negative if the parent does not make cheerful or upbeat bids toward the child (she/he may mutter something occasionally, but it lacks the “bright” quality), and in general appears dutiful rather than genuinely enjoying the “here and now.” Parent may sigh occasionally, look at the clock, etc.

*Code 2 – Discrete Negative*

Code if any of the following affects are present:

- Anger/irritation: irritation in tone or words, scowling, whining due to anger or irritation
- Sad: frowning, pouting, minor injury
- Anxious/fearful: muscle tension, apprehension, worried
- Distressed: generalized mild distress for unknown reasons, well-defined negative affect with unknown cause

*Code 3 – Intense Negative*

Any discrete affect that persists for more than 15 seconds (cumulatively) or affect that occurs several times in one segment (thus, most likely accumulates to more than 15 seconds) should be coded. Any affect that is particularly intense, affect that clearly escalates, or affect that is pervasive and intense should be coded.

Code if any of the following affects are present:

- Anger/irritation: angry yelling, punishment, striking, crying due to anger or irritation
- Sad: tearful, crying
- Anxious/fearful: panicked expression
- Distressed: screaming, crying

**CHILD ANGER CODING SYSTEM (6 CODERS)**

**Latency to anger response Arm Restraint:**

Time, in seconds, starting when parent places hands on C’s arms to the first sign of anger (minimum= 1 sec)

**Latency to anger response Toy Retraction:**

Time, in seconds, starting when parent removes toy from C to first sign of anger (facial, vocalic, postural, or instrumental). The first definite anger response is any response that would be coded as a 1 or higher on the coding sheet (The minimum latency is 1 second.)

**Latency to anger Car Seat:**

Time, in seconds, to the first sign of anger after C is buckled into the car seat.

**Intensity of struggle (Body Anger):**

Peak intensity of struggling in each epoch is rated on the following scale:

0 = No struggling at all. **No resistant** movement.

1 = Low intensity struggle. For instance, 1-2 arm pulls and/or low intensity body movements (e.g. shifting, twitching, light wiggling).

2 = Medium intensity struggle. Sporadically pulls away from parent using arms and/or body- and /or pushes against parent. Movements could include medium intensity pulling of arms, leaning forward, arching back or kicking. Generally 1-2 movements.

3 = Moderately high intensity struggle. Near continuous moderate intensity movements. Can include the same movements as number 2 with higher intensity. Generally 3-4 movements.

4 = High intensity struggle. Continuous movement of moderately high intensity with intervals of high intensity resistance.

**Intensity of anger expression (Facial Anger):**

Presence of anger or anger blends is noted in each epoch using **AFFEX**, and rated on the following scale:

0 = No facial region shows codable anger movement

1 = Only one facial region shows codable movement, identifying a low intensity anger, or expression is ambiguous

2 = Only 2 facial regions show codable movement, or expression in one region (e.g. brows) is definite.

3 = An appearance change occurs in all 3 facial regions, or coder otherwise has impression of strong anger.

**AFFEX Facial Expression Definitions**

**Anger/Rage:**

Brows down straight or slanting down toward the center. Brows are often drawn together. May be bulges or wrinkled around brows. Cheeks should be raised. Mouth could be straight, angular, or drawn tightly shut.

**Intensity of distress vocalizations (Vocal Anger):**

Peak intensity of distress vocalizations is noted in each epoch and rated on the following scale:

0 = No distress

1 = Mild protest verbalization or difficult to identify as hedonically negative

2 = Definite protest, short duration to fussing or mild, low intensity cry (cry has extended or rhythmic quality)

3 = Definite non-muted crying/ full intensity cry/scream (almost loss of control)
